# Concentration-dependent systemic response after inhalation of nano-sized zinc oxide particles in human volunteers

**DOI:** 10.1186/s12989-018-0246-4

**Published:** 2018-02-12

**Authors:** Christian Monsé, Olaf Hagemeyer, Monika Raulf, Birger Jettkant, Vera van Kampen, Benjamin Kendzia, Vitali Gering, Günther Kappert, Tobias Weiss, Nadin Ulrich, Eike-Maximilian Marek, Jürgen Bünger, Thomas Brüning, Rolf Merget

**Affiliations:** 1 Institute for Prevention and Occupational Medicine of the German Social Accident Insurance, Institute of the Ruhr-Universität Bochum (IPA), Bürkle-de-la-Camp-Platz 1, 44789 Bochum, Germany; 2 Gerinnungszentrum Rhein-Ruhr, Königstraße 13, 47051 Duisburg, Germany

**Keywords:** Zinc oxide, Nanoparticles, Concentration-response relationship, Systemic effects, Inhalation study

## Abstract

**Background:**

Inhalation of high concentrations of zinc oxide particles (ZnO) may cause metal fume fever. In an earlier human inhalation study, no effects were observed after exposure to ZnO concentrations of 0.5 mg/m^3^. Further data from experimental studies with pure ZnO in the concentration range between 0.5 and 2.5 mg/m^3^ are not available. It was the aim of this experimental study to establish the concentration-response relationship of pure nano-sized ZnO particles.

**Methods:**

Sixteen healthy subjects were exposed to filtered air and ZnO particles (0.5, 1.0 and 2.0 mg/m^3^) for 4 h on 4 different days, including 2 h of cycling with a low workload. The effects were assessed before, immediately after, and about 24 h after each exposure. Effect parameters were symptoms, body temperature, inflammatory markers and clotting factors in blood, and lung function.

**Results:**

Concentration-dependent increases in symptoms, body temperature, acute phase proteins and neutrophils in blood were detected after ZnO inhalation. Significant effects were detected with ZnO concentrations of 1.0 mg/m^3^ or higher, with the most sensitive parameters being inflammatory markers in blood.

**Conclusion:**

A concentration-response relationship with nano-sized ZnO particles in a low concentration range was demonstrated. Systemic inflammatory effects of inhaled nano-sized ZnO particles were observed at concentrations well below the occpational exposure limit for ZnO in many countries. It is recommended to reassess the exposure limit for ZnO at workplaces.

## Background

Zinc oxide fumes produced by activities such as thermal cutting, welding and melting may induce zinc fever after inhalation. Besides fever, typical symptoms include throat irritation, cough, minor respiratory symptoms, metallic taste, as well as flu-like symptoms, such as a general feeling of illness, myalgia, arthralgia or headache [1]. Typically, the symptoms occur after a latency period of 4 - 12 h, and resolve themselves within 48 h. Tolerance, which may develop after repeated exposures, has been shown to be reversible after several exposure-free days [1]. Generally, zinc fever is assumed to have no long-term sequels, but there is no valid epidemiologic information available.

In previous human experimental inhalation studies, nearly all volunteers developed zinc fever after exposure to 5 mg/m^3^ ZnO for 2 h [2, 3]. Conversely, lower ZnO concentrations of 2.5 mg/m^3^ for 2 h only induced a slight but significant increase in body temperature [3].

Thus far, only one study has analyzed leukocytes in blood after ZnO exposure, with the authors reporting no increase in polymorphonuclear neutrophilic granulocytes (ZnO concentration range 2.76 to 37 mg/m^3^; 15 to 120 min. Exposure duration) [4].

Exposure to 0.5 mg/m^3^ ultrafine and fine ZnO particles produced no effects (symptoms, body temperature, blood and sputum parameters) in 12 subjects after inhalation for 2 h at rest [5].

Earlier studies showed an increase of blood leukocytes about 20 h after exposure to welding fumes of galvanized steel [6, 7]. An asymptomatic increase in high-sensitive C-reactive protein (hsCRP) in the blood of 12 subjects after inhalation of metal inert gas (MIG) welding fumes of zinc-coated steel for 6 h at a zinc concentration of 1.5 mg/m^3^ was reported [8]. In a further study by the same working group, an increase in blood CRP (measured with a high sensitive (hs) ELISA) was detected after exposure to different concentrations of MIG brazing fumes of zinc-coated materials for 6 h. The authors defined a No Observed Effect Level (NOEL) for systemic inflammation for welding fume concentrations (PM_10_) between 1.4 and 2.0 mg/m^3^ (containing 1.1 to 1.5 mg/m^3^ ZnO), but did not rule out that the effects may be different for other processes, even with the same zinc content [9]. Three different welding fumes containing Zn, traces of aluminum with/without copper showed a maximal increase in interleukin-6 (IL-6) 10 h after exposure, but no qualitative differences were observed in inflammatory parameters like CRP and serum amyloid A (SAA), even 29 h after exposure [10].

In summary, experimental inhalation studies investigating zinc containing welding fumes report that zinc-related inflammatory effects occur below 2.0 mg/m^3^ ZnO. However, one study with low concentrations of pure ZnO (0.5 mg/m^3^) reported no effects [5]. Thus, a precise NOEL cannot be defined with the available data, and as a consequence we aimed in the present study to define the concentration-response relationship for pure ZnO in this lower concentration range.

## Methods

### Generation and characterization of ZnO nanoparticles

The principle of the particle synthesis was based on the pyrolysis of atomized aqueous zinc formate solutions with a hydrogen-oxygen flame [11]. The mobility diameter of the generated primary particles was about 10 nm. Depending on the ZnO concentration the primary particles formed aggregates and agglomerates in a range from 48 nm (0.5 mg/m^3^ ZnO) to 86 nm (2.0 mg/m^3^ ZnO). The particle diameters are comparable to those observed in an emission study of galvanized materials with different welding techniques [12]. A ceiling fan was used to homogenize the freshly generated ZnO nanoparticle atmospheres in the exposure unit [13]. Briefly, constant target concentrations with 0.5, 1.0 and 2.0 mg/m^3^ ZnO were planned. Sham exposures (0 mg/m^3^ ZnO) were also performed with the flame generator operated with purified water without zinc salt.

There were negligible differences between target and measured concentrations (Table 1). The particle size distributions were monomodal with relatively small geometric standard deviations of 1.66 to 1.69.

**Table 1:** Measured parameters of airborne ZnO nanoparticles

Target concentration	Measured concentration	Mobility particle diameter	Geometric standard deviation	Particle number concentration
[mg/m^3^]	[mg/m^3^]	[nm]	[GSD]	[#/cm^3^]
0.0	0.016 (+/− 71.8%)	–	–	< 500
0.5	0.514 (+/− 2.4%)	47.8 (+/− 2.4%)	1.66	1.69E + 06 (+/− 3.5%)
1.0	1.013 (+/− 1.1%)	62.8 (+/− 2.7%)	1.68	2.03E + 06 (+/− 4.1%)
2.0	2.014 (+/− 1.2%)	85.8 (+/− 4.0%)	1.69	2.53E + 06 (+/− 9.2%)

The purity of the airborne ZnO was 99.71%. Three precursor solutions were prepared by dissolving 11.0, 22.5 and 46.0 g zinc formate dihydrate (Zn(CH_2_O)_2_ * 2 H_2_O, purity 98%, Alfa Aesar GmbH, Karlsruhe, Germany) in 3.0 mL acetic acid (HAc), which acted as a stabilizer (purity 99%, Merck GmbH, Darmstadt, Germany), and brought to a total volume of 1000 mL with purified water to yield Zn concentrations of 0.057, 0.118 and 0.240 mol/L, respectively. The hydrogen volumetric flow rate was set to 10.0 L/min and the oxygen flow to 5.0 L/min. Argon flow rate was 4.0 L/min. The atomizing gas was nitrogen (nitrogen generator, model NGM 22, cmc instruments GmbH, Eschborn, Germany), and the atomizing pressure was set to 0.30 bar. Typical flow rates of the aqueous zinc formate solutions were in the range of 0.90-1.00 mL/min.

The air exchange rate was set at 12 per hour (360 m^3^/h) with a room temperature of 23.5 °C (+/− 0.3 °C) and a relative humidity of 47.0% (+/− 1.7%).

**Fig. 1:**
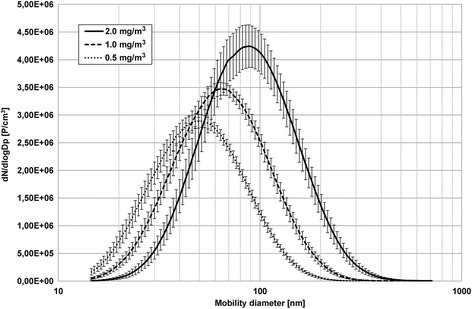
Particle size distributions of airborne ZnO concentrations at 0.5, 1.0 and 2.0 mg/m^3^. In addition, the error bars of each individual size channel are shown

A Scanning Mobility Particle Sizer (SMPS, model 3080, TSI Inc., Shoreview MN, USA, equipped with a long differential mobility analyzer and a butanol condensation particle counter, TSI model 3776) measured the particle size distributions in the exposure unit. The number concentration and size distributions were determined every 5 min. Mass concentration measurements of airborne ZnO were recorded at 1-min intervals using a tapered elemental oscillating microbalance (TEOM, model 1400a, Rupprecht and Patashnik, Albany NY, USA). Trace gas analyses of nitric oxides (NO, NO_2_) were performed via online chemical ionization mass spectroscopy at 1-s intervals (Airsense.net, MS4 GmbH, Rockenberg, Germany) to control the pyrolysis process. All measurement results of airborne ZnO concentrations, mobility particle diameter, geometric standard deviations as well as particle number concentration are listed in Table 1 (averaged over all exposure days for each exposure condition) (Fig. 1).

### Participants

Sixteen healthy nonsmoking volunteers (8 women, 8 men) with a median age of 26 (range 19-42) years participated in the study (Table 2). The subjects had no previous exposure to airborne zinc compounds and did not show bronchial hyperresponsiveness to methacholine as assessed with a reservoir method [14]. Standard baseline laboratory parameters were within normal ranges. Seven subjects sensitized to ubiquitous aeroallergens (atopy screen sx1, Phadiatop, ImmunoCAP system, ThermoFisher Scientific, Phadia AB, Uppsala, Sweden) without any clinical manifestation were included, but exposures were performed outside of the allergy season.

**Table 2:** Characteristics of the study subjects

	Total	Male	Female
	*n* = 16	*n* = 8	*n* = 8
Age [years]	26 (19-42)	28 (19-42)	24 (23-32)
Height [cm]	178 (155-191)	182 (176-191)	164 (155-182)
Weight [kg]	72 (51-104)	83 (61-104)	59 (51-91)
BMI [kg/m^2^]	24 (19-29)	25 (20-29)	23 (19-27)
Total IgE [kU/L]	31 (2-329)	79 (2-208)	28 (20-329)
sx1 [*n* ≥ 0.35 kUA/L]	7	4	3

Medians, minimum and maximum values are listed. BMI = body mass index. Sx1 is an indicator of sensitization to environmental allergens

### Study design

**Fig. 2:**
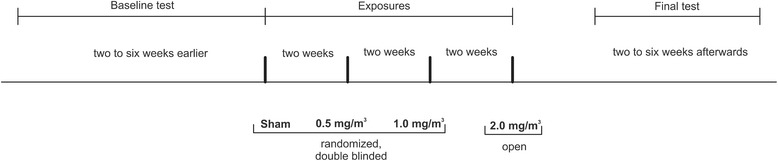
Time line of this study

Exposures were performed in an exposure unit at our institute [15]. Subjects were exposed for 4 h, with 2 weeks intervals for each subject. The subjects were generally at rest, except for short periods of moderate physical activity on a cycle ergometer set to 15 L/min/m^2^ (corresponding to a work load of 60 watt (range 30-96 watt), which was separated into 4 blocks of 30 min each (total 120 min)). Exposures were randomized and double blinded, with the exception of the exposures to 2.0 mg/m^3^ ZnO, which were not blinded according to instructions by the ethics committee. Medical examinations were performed before, directly after, and approximately 24 h after the start of exposure. Additionally, examinations were performed before the first (baseline test) and after the last exposure (final test) (Fig. 2).

At each examination, subjects answered a questionnaire and underwent a physical examination, blood sampling, lung function testing, and measurements of fractional exhaled nitric monoxide (FeNO) and body temperature. In addition, vital functions (electrocardiogram, blood pressure) were monitored during the exposures.

### Questionnaire

All subjects answered a questionnaire addressing flu-like symptoms (at least 1 of 3 symptoms: feeling of fever, feeling sick and muscle pain), and additionally all symptoms were graded according to severity (not at all (1 score point), barely (2 points), little (3 points), moderate (4 points), strong (5 points), very strong (6 points)). To avoid any information bias we added questions about itching nose, abdominal pain, metallic taste in mouth, dry nose, throat irritation, headache, cough, burning eyes, feeling warm, itching skin, nausea, shortness of breath, dry eyes, sweating, irritation of the eyes, general irritability, feeling cold, chills, runny nose, feeling unwell, bleeding eyes and muscle cramps.

### Body temperature

Subjects measured their own body temperatures using an infrared method that recorded the temperature in both ears. This was performed at each examination, and at intervals of 2 h from the beginning of the exposure to 24 h afterwards, but not during sleep (Braun Thermoscan Pro 4000, WelchAllyn, Hechingen, Germany).

### Blood parameters

Markers of inflammation (blood cell counts, CRP, SAA, Club cell protein (CC16), IL-6) and coagulation (prothrombin F 1.2, endothelial microparticles, fibrinogen, D-dimers) were analyzed. The total and differential blood cell counts were determined using standard procedures with UniCell DxH800 (Beckman Coulter Inc., Brea CA, USA). Sandwich ELISA were used to measure CC16 (BioVendor, Brno, Czech Republic, range 1.57-50 ng/mL), IL-6 (R & D Systems, Wiesbaden, Germany, range 4.7-600 pg/mL), SAA (Invitrogen™ Novex ™ SAA Human ELISA Kit which detect serum amyloid A1 cluster, Carlsbad CA, USA, range 9.4-600 ng/mL), and CRP (high sensitive ELISA from Siemens Healthcare Diagnostics Products GmbH, Marburg, Germany, precision levels down to or below 0.3 mg/L) in serum.

The clotting factors were measured in plasma according to the manufacturer’s instructions: Prothrombin cleavage fragment F1.2 with Enzygnost F1.2 (Siemens, range 69-229 pmol/L), endothelial microparticles with ZYMUPHEN MP (Hyphen Biomed, Neuville-sur-Oise, France, precision levels down to or below 5 nM), fibrinogen with a modified method from Clauss with Multifibren U (Siemens, range 180-350 mg/dL), and D-dimers with Behring Coagulation System XP (BCS XP) and INNOVANCE D-Dimer (Siemens, precision levels down to or below 0.5 mg/L).

### Biomonitoring

Biomonitoring was performed by determination of zinc levels in blood and urine. Plastic materials were used for sample preparation to prevent contamination. Prior to usage, the vessels were cleaned with 1% nitric acid for 2 h, rinsed with ultrapure water and dried at room temperature. After thawing the frozen aliquots, 50 μL of the blood samples were diluted 1:100, and 500 μL of the urine samples were diluted 1:10 with 0.1 M HCl solution and 100 μL of a 0.2% solution of Triton-X. Rhodium was used as internal standard. Analysis was carried out using a 7700 ICP-MS system from Agilent Technologies in He-mode (flow rate 5 mL/min) with a collision cell to avoid interferences. Skimmer and sampler cones were made of nickel. Calibration and calculation of the Zn concentrations were carried out using standards at eight different concentrations. LOQ was 4.0 μg/L. Materials from RECIPE (ClinChek Whole Blood Level, lyophil. For Trace Elements I and II, REF 8840, LOT 227; ClinChek Urine Control lyophil. For Trace Elements I and II, REF 8847-8849, LOT 122) and SERONORM (Trace Elements Whole Blood Level I and II, LOT 1103129, Trace Elements Urine Level I and II, LOT 1011644) served as internal control.

### FeNO

Fractional exhaled nitric oxide was measured using a portable electrochemical analyzer (NIOX Mino, Aerocrine, Solna, Sweden) taking into account the guidelines of the American Thoracic Society and European Respiratory Society [16].

### Lung function testing

Lung function was recorded using both body plethysmography [17] and spirometry [18] in a linked maneuver with a MasterLab (Jaeger, Würzburg, Germany). A battery of different parameters was evaluated (e.g. airway resistance, lung volumes, and flows).

### Data analysis

Descriptive analysis was performed for each variable stratified by exposure (sham, 0.5, 1.0 and 2.0 mg/m^3^ ZnO) and time of measurement (before, during, immediately after, and 24 h post exposure). Characteristics of subjects were expressed as medians, 25%- and 75%-quantiles, as well as minimum and maximum. Graphical representations were illustrated with boxplots. Effects were compared between the before, immediately after, and 24 h after exposure. In addition, the effects after sham were compared to exposure after ZnO. Exposure groups were compared using paired Student’s t-test for normal or log-normal distributed variables. The problem of multiple comparisons was counteracted using the Bonferroni correction, by dividing the overall desired statistical significance level α = 0.05 by the number of hypotheses tested. Individual descriptive analyses were performed for body temperature with a cut-off of ≥37.5 °C. Spearman correlation coefficients (r_S_) were calculated to predict the monotone association between parameters.

Rank order tables were developed to give another estimate of increased effects which follow a concentration-response relationship. Increased effects were defined as values bigger than the largest value of baseline test, examinations before each exposure and final test (*n* = 6) plus the double median absolute deviation (MAD) of these 6 values (> max (baseline test, final test, initial investigation before start of exposure) + 2 MAD). Each of the ZnO related effects was assigned to ranks 1 to 4, the lowest value represented by rank 1 and the highest by rank 4, respectively. All parameters were evaluated with both group comparisons and rank order tables.

## Results

The results obtained from baseline tests, examinations done before each exposure, and from final tests (*n* = 6) were not significantly different from one another. In addition, when the final tests were conducted at the end of the study (minimum 14 days after the last exposure), all parameters had returned to levels that were within the range of the initial values. Consequently, only those parameters with significant changes are presented below. ZnO exposure had no effect on CC16, IL-6, clotting factors (prothrombin F 1.2, endothelial microparticles, fibrinogen, D-dimers) in blood, FeNO, and all lung function parameters.

### Questionnaire

**Fig. 3:**
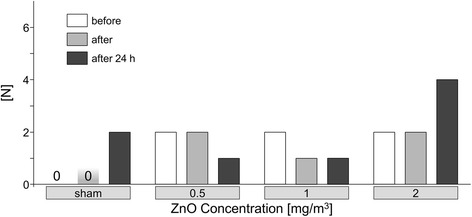
Number of subjects with flu-like symptoms (at least 1 of 3 symptoms: feeling of fever, feeling sick and muscle pain) according to ZnO concentrations and time points

There was a small increase in the number of subjects with flu-like symptoms 24 h after exposure to 2.0 mg/m^3^ ZnO when compared to the number of complaints directly after exposure (Fig. 3). The same results were also obtained if the grading of severity of symptoms was considered (data not shown). Also there were no concentration-related effects with any other questions (data not shown).

### Body temperature

With the exception of one male subject who suffered from an abdominal infection during the sham exposure, there was no significant increase in body temperature (≥ 37.5 °C) in subjects exposed to either sham or 0.5 mg/m^3^ ZnO. Only two subjects exposed to 1.0 mg/m^3^ ZnO reported increased temperatures of ≥37.5 °C; whereas, six subjects had elevated temperatures upon exposure to 2.0 mg/m^3^ ZnO. Furthermore, only one subject reported increased body temperature after exposure to both 1.0 and 2.0 mg/m^3^ ZnO. All temperature increases occurred between 8 to 10 h post exposure, with the maximum reported temperature of 39.5 °C in females and 38.6 °C in males. Importantly, the increase in temperature in all subjects was not significantly different within group comparisons.

### Blood parameters

**Fig. 4:**
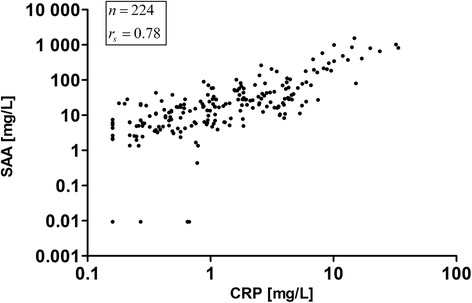
Correlation plot of SAA vs. CRP. All values (*n* = 224) at all ZnO concentrations are shown

The concentration of the acute phase proteins CRP and SAA were highly correlated (r_S_ = 0.78) as illustrated in Fig. 4. The Spearman correlation coefficient of r_S_ = 0.78 was calculated including all values at all exposure conditions. Coefficients for each concentration yielded similar results (0 mg/m^3^ ZnO: r_S_ = 0.68, 0.5 mg/m^3^ ZnO: r_S_ = 0.86, 1.0 mg/m^3^ ZnO: r_S_ = 0.74 and 2.0 mg/m^3^ ZnO: r_S_ = 0.89).

**Fig. 5:**
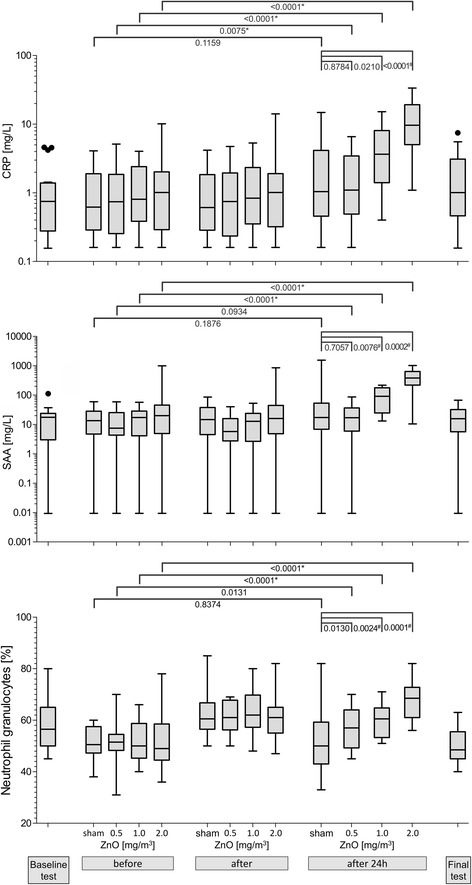
Selected blood parameters according to ZnO concentrations and time points. *significant values with significance level α = 0.0125 (after Bonferroni correction). ^#^significant values with significance level α = 0.0167 (after Bonferroni correction). Outliers are defined as values above median + 1.5 x interquartile range or values below median − 1.5 x interquartile range

Exposure to ZnO led to a concentration-dependent increase in both CRP and SAA levels in the blood 24 h after exposure. When compared to the levels before exposure, blood CRP levels significantly increased with all ZnO concentrations 24 h after exposure. Conversely, SAA levels increased 24 h after exposure to 1.0 and 2.0 mg/m^3^ ZnO (Fig. 5). Compared to the sham exposure, ZnO exposures yielded significantly higher CRP values 24 h after exposure to 2.0 mg/m^3^ ZnO, and higher SAA values after 1.0 and 2.0 mg/m^3^ ZnO (Fig. 5).

Absolute and relative numbers of neutrophil granulocytes were similar to each other and as a result only the relative numbers were shown as a percentage (Fig. 5c) (absolute numbers not shown). In contrast to the acute phase proteins, neutrophil levels increased significantly immediately after all exposure scenarios (including sham), but not in a concentration-dependent relationship, suggesting an effect of the physical exercise [19]. A concentration-response was observed only after 24 h after exposure. Neutrophil levels increased 24 h after exposure to 1.0 and 2.0 mg/m^3^ ZnO compared to levels before the exposure (Fig. 5). Furthermore, compared to the sham exposure, all concentrations of ZnO elicited significant increases in neutrophils 24 h later (Fig. 5).

Four subjects showed increased CRP levels upon exposure to sham exposures as illustrated in the rank order tables, including the one subject (ID 3) with an acute abdominal infection (Table 3). The effect increased with increasing ZnO concentrations with 6 subjects (CRP) after 0.5 mg/m^3^ ZnO, 12 subjects (CRP and SAA) after 1.0 mg/m^3^ ZnO, and up to 15 subjects (CRP) after 2.0 mg/m^3^ ZnO. The acute phase proteins (CRP and SAA) increased in a concentration dependent manner after exposures to 1.0 and 2.0 mg/m^3^ ZnO. In contrast, neutrophils and body temperature significantly increased only after 2.0 mg/m^3^ ZnO. No susceptible subjects were identified, because increased effects occurred in all subjects, without increased frequency in single subjects (Table 3).

**Table 3:**
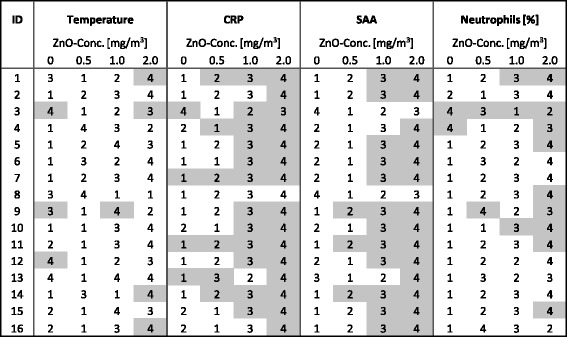
Rank order tables

Grey colored cells represent increased effects defined as > max value of baseline test, final test and initial investigation before start of exposure plus double median absolute deviation (MAD) of these 6 values. Numbers indicate ranks of effects

There were no differences between males and females, as well as between subjects with and without sensitizations to environmental allergens (data not shown). Levels of Zn in blood and urine were unaffected by ZnO inhalation at any concentration (data not shown). This is due to the naturally occurring high zinc concentrations in the blood, which are in the range between 4 to 7.5 mg/L whole blood [20]. In addition, the concentration shows large fluctuations, influenced by food intake, but the diet of our subjects was not standardized in this study.

### Calculation of deposition rates of inhaled ZnO particles

We used the ICRP model [21] to identify possible differences of deposition rates of ZnO particles with different median diameters in the tracheobronchial and alveolar region. Due to the way of particle generation, the median diameter of the ZnO particles at 0.5 mg/m^3^ with 47.8 nm increases at 1.0 mg/m^3^ ZnO to 62.8 nm and at 2.0 mg/m^3^ ZnO to 85.8 nm. The estimations of the deposition rates were performed under the following conditions: The ZnO particles are ideal spheres, the measured mobility diameter corresponds to the activity median aerodynamic diameter in the ICRP model and the density of the particles is 1 g/cm^3^. We used the data of the “Reference Worker” in this model. Table 4 shows the estimation of deposition rates in dependence of different particle size and lung compartments.

**Table 4:** Estimated depostion rates of ZnO in the current study

Particle concentration	Median particle diameter	ZnO deposition rate	ZnO deposition rate
[mg/m^3^]	[nm]	(sum of tracheobronchial,	(alveolar)
		inhalable and alveolar)	
0.5	47.8	78%	48%
1.0	62.8	74%	47%
2.0	85.8	66%	44%

## Discussion

Experimental inhalation studies in humans with nanoparticles, which use multiple concentration steps in order to describe a concentration-response relationship are extremely sparse. The present study is, to our knowledge, the first human inhalation study with pure nano-sized ZnO in the concentration range of 0.5 to 2.0 mg/m^3^. A key result of this study is the demonstration of such a concentration-response relationship. The most sensitive outcomes were the increase in acute phase proteins (CRP and SAA) and neutrophils in blood, followed by an increase in body temperature and the occurrence of flu-like symptoms at and above 1 mg/m^3^ ZnO.

No relevant effects were detectable after sham exposures, but initial increases of acute phase proteins were observed with a concentration of 0.5 mg/m^3^ ZnO after 24 h. Pronounced effects on acute phase proteins and neutrophils occurred after 1.0 mg/m^3^ ZnO, together with increases in body temperature in some subjects. All the effects were strongest after 2.0 mg/m^3^ ZnO, with flu-like symptoms and elevated body temperature measured in several subjects.

The results of this study are in accordance with previous studies that reported effects (flu-like symptoms, increase in temperature and inflammatory markers) after exposure to ≥2.5 mg/m^3^ ZnO [2–4]. The only previous study that investigated lower concentrations, i.e. 0.5 mg/m^3^ ZnO [5] showed no effects, but importantly, the duration of exposure was only 2 h without physical exercise, resulting in an approximately 4 fold lower inhaled dose (with linear extrapolation) of ZnO in comparison with our lowest ZnO dose. The findings of the present study are also compatible with a previous study investigating ZnO-containing welding fumes [8], which showed an increase in CRP levels after 6 h exposure to 1.5 mg/m^3^ zinc with short periods of physical exercise.

Our investigations were conducted with ZnO as a single component in the nanoscale range at maximal concentrations considered to represent high workplace exposures [12]. The observed effects in this study were caused only by the different airborne ZnO concentrations. Any influences from secondary components of welding fume and trace gases are negligible. Possible effect differences due to different deposition rates of ZnO particles with concentration-dependent median diameters (see Table 1) can be nearly excluded. Our estimation with the help of the ICRP model revealed similar deposition rates of the different particle sizes in this study in terms of both total deposition (sum of alveolar, inhalable and tracheobronchial) and alveolar deposition alone (see Table 4).

The effects measured in this study are indicative of systemic inflammation which may be explained by either primary local inflammation of the respiratory tract/lung and secondary resorption of inflammatory markers, or by primary systemic inflammation due to resorbed zinc ions. The latter mechanism is supported by the 4.8 to 19.2 h half-life of ZnO particles in rat lungs [22, 23]. In addition, Cho et al. [24] hypothesized that the rapid pH-dependent dissolution of ZnO particles inside phagosomes is the main cause of ZnO-induced diverse progressive lung injuries. It has been shown in an ex-vivo study that the release of Zn ions from ZnO nanoparticles, due to their instability in the acidic compartment of lysosomes, increases reactive oxygen species generation and damage of lysosomal membranes and activation of executioner caspase-3 and caspase-7 [25]. The ZnO-induced effects strongly argue for a zinc-specific mechanism, since the distinct clinical presentations of metal fume fever by ZnO as well as other metal compounds, have not been observed after exposure to poorly soluble particles (PSP) [26].

While the acute phase proteins in blood clearly increased after ZnO inhalation, effects on further inflammatory parameter in the blood, such as IL-6 and the secreted CC16 as of a marker of increased lung permeability could not be detected and thus a complete inflammatory response could not be observed in the blood samples obtained at the selected time points. Very recently, it was shown that IL-6 increased 10 h after the start of a 6 h exposure to zinc-containing welding fumes, but this increase was not seen 29 h after the start of exposure [10]. Thus, it is probable that an increase in IL-6 concentration in the blood was overlooked due to the time intervals of the blood sampling in the present study. The increase in body temperature in a subset of subjects suggests susceptibility. Interestingly, increases in inflammatory markers were observed in all subjects - a finding that does not support the concept that only susceptible individuals are affected.

Generally, chronic effects cannot be derived from acute human inhalation experiments. The observation that zinc fever occurs predominantly on the first day after periods without exposure to zinc-containing fumes suggests poorly understood adaptation processes. Zinc fever is proposed to be an acute disease, but epidemiologic information on the long-term consequences of exposure is limited. Our data clearly demonstrate that all observed effects were reversible at the time of the final tests.

Inflammatory systemic biomarkers have been proposed as biomarkers of cancer [27, 28], heart diseases [29–31] and chronic obstructive pulmonary disease and comorbidities [32] in epidemiological studies. However, it is unknown whether the association between inflammatory markers and disease is causal. A number of controlled studies in humans report increased levels of CRP or SAA after particle inhalation, and it is believed that this acute phase pulmonary and systemic response following particle inhalation may indeed constitute a causal link between particle inhalation and for example cardiovascular disease [33]. Very recently, it was shown for the first time that anti-inflammatory therapy led to a significantly lower rate of recurrent cardiovascular events than placebo [34]. This finding strengthens the view that inflammation is indeed causal, thus prevention of cardiovascular disease should comprise, among other measures, minimization of particle inhalation.

It has been reported earlier that pulmonary inflammatory responses following zinc oxide particle inhalation were much stronger than after inhalation of PSP like magnesium oxide. Thus particle composition is a major contributor to inflammation [26]. Epidemiological studies with cancer and cardiovascular disease as endpoints do not exist in predominantly ZnO exposed occupational cohorts. A dose-dependent association between welding fumes and lung cancer has been described for welders [35], and there are weak indications that welding fumes are a risk factor for cardiovascular diseases [36, 37]. The inflammatory acute response after ZnO inhalation in our study was reversible within a few weeks. It remains to be assessed whether repeated or chronic exposure to ZnO increase the risk for diseases which have been shown to be associated with environmental particle inhalation.

One weakness of the present study is that the effect parameters were recorded at limited time points, suggesting that some effects were overlooked due to their shorter or longer kinetics. However, the time points of the increases in the most sensitive parameters - CRP and SAA - in this study correspond well with other evaluations [10]. A further weakness is the lack of blinding of the highest ZnO concentration. In general, inhalation studies should be blinded, but for security reasons due the high ZnO concentrations, this was not performed. However, confounding of objective inflammatory markers in blood should be negligible.

For most of the effect parameters in this study, reference values do not exist or show large inter-individual variability. Thus, with the exception of body temperature (with well-known reference values), descriptive analyses with respect to reference values and interpretations of the magnitudes of the effects were not considered. In this study, two different group comparisons (before / after exposure; sham / ZnO) and additional intra-individual analyses by rank order tables were used. The latter were developed in order to overcome the problem of multiple testing and associated definition of significant effects. Here six control scenarios were available, thus accidental variabilities were minimized. A strength of this study is the lack of effects after sham exposures, with the exception of one subject with an abdominal infection, as well as rare and minor unexplained increases of CRP (but not SAA) in three females. The lack of increased effects in sham exposures, and the consistent concentration-related effects in almost all subjects add to the credibility of the study results.

## Conclusions

In summary, this study was able to demonstrate a concentration-response relationship of ZnO nanoparticles with clear systemic effects at and above 1 mg/m^3^ ZnO. The results are in accordance with previous experimental studies that showed no effects at 0.5 mg/m^3^ ZnO [5] and clear effects concerning CRP with concentrations between 1.1 and 1.5 mg/m^3^ ZnO-containing welding fumes [9]. Similarly an increase of SAA was observed after inhalation of zinc or copper containing welding fumes [38], but the different exposure scenarios within these studies have to be considered. A No Effect Exposure Level (NOEL) derived from our study would be defined between 0.5 and 1 mg/m^3^, although, in contrast to a previous study with 2 h exposures at rest [5], initial effects were seen with ZnO exposures of 0.5 mg/m^3^ concerning CRP and SAA, which were the most sensitive parameters. Exposure limits of ZnO in many countries (e.g. USA, United Kingdom, Australia, Canada) were set to 5.0 mg/m^3^ [39]. As this and more recent experimental studies showed effects far below this concentration, it is highly recommended to reassess the exposure limit for ZnO at workplaces.

## Abbreviations

BMI: Body mass index
CC16: Club cell protein
CRP: C-reactive protein
FeNO: fractional exhaled nitric monoxide
GSD: geometric standard deviation
IL-6: interleukin-6
MAD: median absolute deviation
MIG: metal inert gas welding
NO: nitrogen monoxide
NO_2_: nitrogen dioxide
NOEL: No observed effect level
PM_10_: Particulate matter with an aerodynamic diameter smaller than 10 μm
PSP: Poorly soluble particles
r_S_: Spearman correlation coefficients
SAA: Serum amyloid A
SMPS: Scanning mobility particle sizer
TEOM: Tapered elemental oscillating microbalance
ZnO: Zinc oxide
